# Optic Neuritis Presented as Syndrome of Inappropriate Antidiuretic Hormone Secretion in an 8 Year Old

**DOI:** 10.1155/2021/6672827

**Published:** 2021-03-16

**Authors:** T. G. M. Prasadani, Kapila Panditha, D. Irugalbandara

**Affiliations:** ^1^Pediatric Ward, District General Hospital Avissawella, Avissawella, Colombo, Sri Lanka; ^2^Ophthalmology Unit, Lady Ridgeway Hospital for Children, Colombo, Sri Lanka

## Abstract

Optic neuritis is a rare demyelinating disorder, which involves the optic nerve. It can be a monophasic self-limiting illness due to postinfectious or postvaccination etiology. It can also be an initial presentation of a relapsing demyelinating disorder such as multiple sclerosis or neuromyelitis optica spectrum of disorders. It is characterized to aquaporin-4 antibody-rich areas in the brain, optic nerve, and spinal cord. The hypothalamus and periventricular area are also rich in specific antibodies and may lead to dysfunction in the hypothalamic-pituitary axis. Antidiuretic hormone (ADH) is synthesized in the hypothalamus and stored in the posterior pituitary and may secrete inappropriately due to this disturbance. This will impair water excretion from the kidney, leading to hyponatremia. When hyponatremia is significant, the patient will present with confusion, agitation, and convulsions. This case report discusses acute symptomatic hyponatremia as the initial presentation of optic neuritis due to syndrome of inappropriate ADH secretion (SIADH).

## 1. Introduction

Optic neuritis usually presents with eye-related symptoms. We present an 8-year-old girl, who presented with acute symptomatic hyponatremia due to SIADH. Within 12 hours, she developed acute loss of vision. Optic neuritis was confirmed with retrobulbar involvement in ophthalmologic examination and prolonged latency in visual evoked potential. MRI of the brain and optic tract was inconclusive due to the difficulty in performing postgadolinium enhancement.

The child dramatically improved with corticosteroids, but still, the left eye is having prolonged latency even after 8 weeks of the initial presentation. It could be evolving neuromyelitis optica spectrum of disorders, which may relapse later in the disease course.

## 2. Case History

An 8-year-old previously healthy girl presented to a base hospital in Sri Lanka, and she has had watery diarrhea for 2 days followed by several bouts of vomiting. There was a febrile proceeding upper respiratory illness in the recent past (2 weeks) noted.

There was no drug or toxin exposure to be noted.

On presentation, she developed a generalized tonic-clonic seizure, which lasted for 2 minutes. Following the convulsion, she was confused, agitated, and was difficult to control.

Her initial serum sodium was 125 mmol/l, serum osmolality was 256 mmol/l, urinary osmolality was 356 mmol/l, and urinary sodium was 55 mmol/l. Her hematocrit was normal. Serum albumin was 4.5 g/dl.

She was hemodynamically normal, and there were no features of dehydration. Her sodium was corrected with 3% NaCl 3 ml/kg as a bolus over 20 minutes followed by 0.9% NaCl maintenance. Her sodium took three days to normalize with sodium supplementation, and SIADH did not recur. Her serum electrolytes were monitored closely. She improved clinically with settlement of drowsiness, confusion, and agitation.

On first day afternoon, she lost her vision of bilateral eyes suddenly. There were no other neurological symptoms evident. Her complete neurological examination was normal, other than vision. Color vision was completely absent in both eyes (0/14). Bilateral pupillary reaction had relative afferent pupillary defect. Her visual acuity was impaired; in both eyes, it was 6/9.

**Figure 1:**
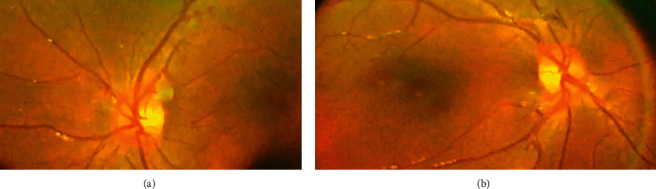
Normal fundus photograph of the (a) right and (b) left eye.

Her ophthalmologic evaluation of the right and left eyes was normal as shown in Figures 1(a) and 1(b), respectively.

Her bilateral visual evoked potential was delayed. All these findings together clinically confirmed bilateral retrobulbar neuritis.

MRI of the brain, spine, and optic tracts was done, but gadolinium enhancement was not performed as her serum creatinine was high. The patient was given IV acyclovir assuming it to be meningoencephalitis initially, and the patient developed acute kidney injury secondary to acyclovir therapy.

**Figure 2:**
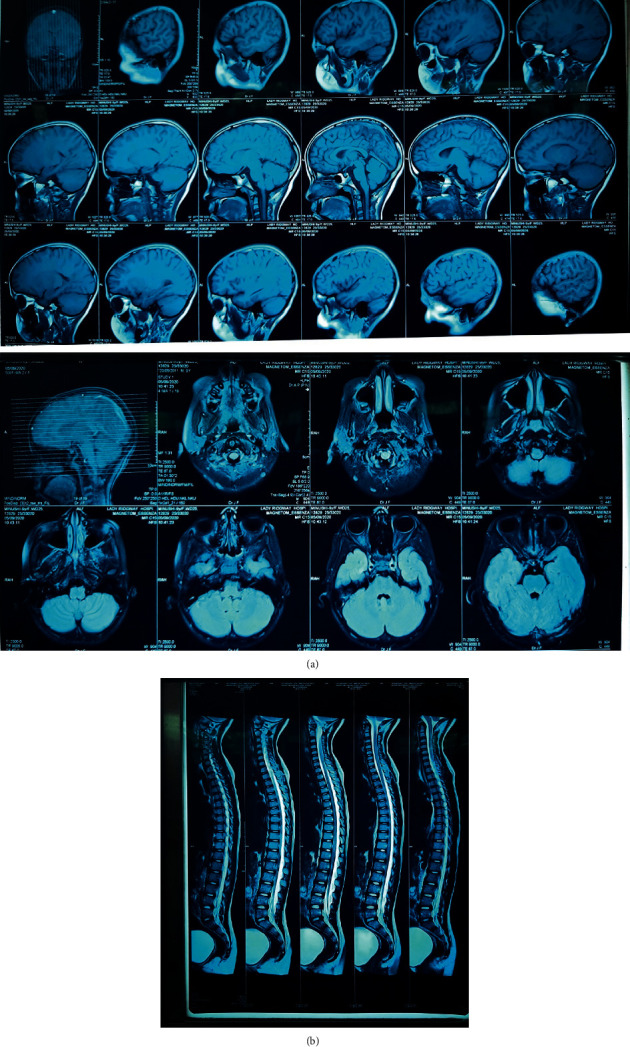
Normal MRI of the (a) brain and optic tract without gadolinium enhancement and (b) spine without postgadolinium enhancement.

The MRI findings of the brain (Figure 2(a)) and spine (Figure 2(b)) were normal.

Her complete blood count was normal, ESR was 61 mm/1^st^ hour, and CRP was <5. Antinuclear antibodies (ANA) for CMV (cytomegalovirus), EBV (Epstein–Barr virus), VZV (varicella-zoster virus), and mycoplasma were negative. Rheumatoid factor was negative.

CSF full report was normal, protein level was 24 mg/dl, glucose level was 5 mg/dl, and no cells were detected. CSF HSV (herpes simplex virus) 1, 2 PCR, EBV, CMV, measles, mumps, and rubella were negative. CSF tuberculosis culture and GeneXpert study were negative. CSF oligoclonal bands were negative. Anti-aquaporin-4 antibody (AQP4-Ab) and anti-myelin oligodendrocyte antibody (MOG) were negative.

She was diagnosed with optic neuritis.

Intravenous methyl prednisolone 30 mg/kg/day was given for 3 days followed by oral prednisolone which was tapered gradually.

Her clinical symptoms improved dramatically with IV methyl prednisolone, and on day 3 of commencement, she regained her normal visual acuity. Her color vision was completely restored in one week time. Her ESR was 21/1^st^ hour after 2 weeks.

**Figure 3:**
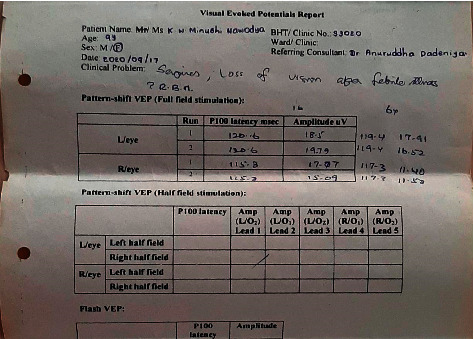
Visual evoked potential after 2 weeks of the acute event.

However, the repeat visual evoked potential after 8 weeks still prolonged in the left eye and was normal in the right eye (Figure 3).

Throughout the illness up to now, she did not develop any other neurological manifestations.

## 3. Discussion

Optic neuritis is an acquired demyelinating disorder in the central nervous system [1, 2].

It has a postinfectious or postvaccination etiology most of the time, but it could be the first presentation of multiple sclerosis and neuromyelitis optica spectrum of disorders (NMOSD) [1–3].

It can be secondary to systemic inflammatory conditions (SLE and sarcoidosis), mitochondrial disorders (Leber hereditary optic neuropathy), toxins (etanercept), ischemia, and compressive neuropathy (primary tumors, metastasis, and tuberculoma) [1].

It is usually sudden in onset and presents as acute unilateral/bilateral blindness, color vision impairment (dyschromatopsia), impairment of unilateral/bilateral visual acuity, painful eye movements, and relative afferent pupillary defect [1, 3].

Acute hyponatremia due to syndrome of inappropriate secretion of antidiuretic hormone (SIADH) is a rare association of NMOSD and optic neuritis-associated NMOSD [4, 5].

SIADH is a disease entity where dysfunction of the hypothalamic-pituitary axis leads to excessive secretion of the antidiuretic hormone (ADH) or ADH-like substances [5].

AQP4-Ab is found in the brain throughout, but its expression is quite rich in the optic nerve and spinal cord, making patients more susceptible to develop optic neuritis and NMOSD [5].

Its expression is abundant in the hypothalamus and periventricular area also; hence, dysfunction of these brain structures can give rise to SIADH [5].

The presence of AQP4-Ab and MOG-Ab is supportive for the diagnosis of optic neuritis or evolving NMOSD, but mere absence does not disregard the diagnosis. There is an entity described as seronegative NMOSD, where the aforementioned antibodies are negative [6].

SIADH will lead to impaired water excretion. Therefore, the patient will have dilution of intravascular volume, resulting in hyponatremia. There will be an expansion of extracellular volume as a result of water retention. The kidney will attempt to excrete sodium, and urinary sodium will increase. Urine osmolality will be > 100 mosm/kg, serum osmolality will be < 280 mosm/kg, and urine sodium will be >30 mmol/l.

Since it is euvolemic hyponatremia, no features of dehydration or shock will be encountered.

In this case, it was a previously normal child presenting with symptomatic hyponatremia with generalized convulsion, confusion, and agitation, but was euvolemic.

After the correction of hyponatremia, her general condition improved, and acute visual loss developed in 12 hours. It was diagnosed to be optic neuritis, with suggestive ophthalmologic examination and VEP, but the MRI of the brain, spinal cord, and optic tracts was inconclusive without postgadolinium enhancement.

The patient improved dramatically with intravenous corticosteroids, and her subsequent serum sodium levels were also normal.

However, she still has prolonged latency of her left eye in her latest VEP (Figure 3).

It is difficult to comment at this stage whether this is monophasic optic neuritis or evolving NMOSD. She needs regular follow-up in the disease course.

There are very few published cases which discussed the association of SIADH and NMOSD. Reported pediatric cases are even rare.

This case highlights the possibility of optic neuritis or evolving NMOSD, presenting with an unusual presentation of acute symptomatic hyponatremia due to SIADH.
